# Comparative Analysis of Deep Learning Models Used in Impact Analysis of Coronavirus Chest X-ray Imaging

**DOI:** 10.3390/biomedicines10112791

**Published:** 2022-11-02

**Authors:** Musiri Kailasanathan Nallakaruppan, Subhashini Ramalingam, Siva Rama Krishnan Somayaji, Sahaya Beni Prathiba

**Affiliations:** 1 School of Information Technology and Engineering, Vellore Institute of Technology, Vellore 632014, India; 2 School of Computer Science and Engineering, Vellore Institute of Technology, Chennai 600127, India

**Keywords:** CNN, CT, X-ray, MRI, COVID-19, resnet, VGG16, Xception, InceptionV3

## Abstract

The impact analysis of deep learning models for COVID-19-infected X-ray images is an extremely challenging task. Every model has unique capabilities that can provide suitable solutions for some given problem. The prescribed work analyzes various deep learning models that are used for capturing the chest X-ray images. Their performance-defining factors, such as accuracy, f1-score, training and the validation loss, are tested with the support of the training dataset. These deep learning models are multi-layered architectures. These parameters fluctuate based on the behavior of these layers, learning rate, training efficiency, or over-fitting of models. This may in turn introduce sudden changes in the values of training accuracy, testing accuracy, loss or validation loss, f1-score, etc. Some models produce linear responses with respect to the training and testing data, such as Xception, but most of the models provide a variation of these parameters either in the accuracy or the loss functions. The prescribed work performs detailed experimental analysis of deep learning image neural network models and compares them with the above said parameters with detailed analysis of these parameters with their responses regarding accuracy and loss functions. This work also analyses the suitability of these model based on the various parameters, such as the accuracy and loss functions to various applications. This prescribed work also lists out various challenges on the implementation and experimentation of these models. Solutions are provided for enhancing the performance of these deep learning models. The deep learning models that are used in the prescribed work are Resnet, VGG16, Resnet with VGG, Inception V3, Xception with transfer learning, and CNN. The model is trained with more than 1500 images of the chest-X-ray data and tested with around 132 samples of the X-ray image dataset. The prescribed work analyzes the accuracy, f1-score, recall, and precision of these models and analyzes these parameters. It also measures parameters such as training accuracy, testing accuracy, loss, and validation loss. Each epoch of every model is recorded to measure the changes in these parameters during the experimental analysis. The prescribed work provides insight for future research through various challenges and research findings with future directions.

## 1. Introduction

The universal spreading and mutation of the COVID-19 pandemic has resulted in havoc, with the loss of millions of lives. The fundamental issue is that the clinical and medical care divisions are researching this epidemic spread. As a result, it is necessary to consider the study of the hypothetical situation which is not limited to working with the next step for the sufferers, but also for the reduction of the tainted persons. The MRI assessment approach is the most common one because it is cost effective, speedy and has no constraints with the space issues.

It plays a vital part in corona patient screening and illness strength detection. MRI scans can help us to evaluate the strength of the lungs of the patient since the coronavirus attacks the respiratory epithelial cells. The most efficient approach for detecting these components through MRI has been an area of concern. The convolution of the brain neural network made significant progress in recognizing the images, notably with realm of assistive clinical decision-making innovation. Brain networks have been successfully applied to identify viral fever from MRI, with achievement of the exhibitions that are superior to those of the radiologists. We are segregating COVID-19 instances from non-COVID-19 cases using the neural network models. The primary goal is to use a profound model for achieving better precision of arrangement using X-ray and CT images. With the objective of analysis, there are huge datasets of the COVID-19 scan reports, and X-rays are available on the internet. The database was obtained from the internet source for the evaluation of this problem. The training and test samples were prepared from the datasets with separate databases. COVID-19 and non-COVID-19 people’s chests images are included in both databases. The X-ray database has 67 entries. The COVID-19 dataset has 345 COVID-19 images with a comparable quantity of non-COVID-19 images, but the CT filter dataset has 345 COVID-19 images with a similar quantity of non-COVID-19 images. To begin the evaluation, images are resized to an aspect ratio of 50:50 from their original size. To assess the model’s adequacy, the irregular sub-sampling or holdout approach is used. In addition, this result shows higher consistency when layers in the CNN-based deep model are altered. Various evaluation metrics, such as accuracy in training and testing and loss measured due to training and validation, were used to vigorously and meticulously examine the system. The advantages of these measurements are not completely resolved on varied proportions of preparing and testing data, considering distinct layers in the in-depth model for better accuracy.

### 1.1. Existing System

The radiologist critically examines the X-ray images and presents the reports to the specialist. The arrangement of the radiographer is the time-consuming task, and also the reports may be deferred. Because they are finished manually with human intervention, at some point, manual mistakes are prone to occur while composing the reports, and also the delay in the reports could create major issues pertaining to the diagnostic process. Furthermore, the experimental efforts of the radiologist would be extremely difficult, and also the results obtained may not be accurate.

#### Demerits of the Existing Systems

- Requirement of a profound radiologist to propose the MRI result.
- If the radiologist is not available, then the postponement of the report could lead to further complications.
- The medical clinic needs to physically have the radiologist present, and this leads to additional costs in the treatment process.

### 1.2. Proposed System

The proposed system makes use of the CT report of the COVID-19 admissions. Medical practitioners diagnose these medical images using their clinical processing and interpretation. Different physicians give different analyses and interpretations of these scanned images with different clinical interpretations. Moreover, images which are acquired at different time slots are much harder to predict in one shot. Additionally, the responsibility of the translation of a med-image is extremely hard. which leads to exhaustion for the medical practitioners working in this regard. With this scenario, we have the highest priority requirement on information and communication technology (ICT) for the fastest and superior prediction of the disease and timely evaluation and generation of the reports. In order to achieve this, we need image classification for the study of COVID-19 images to predict the disease faster and more accurately using the deep image classification methods. The proposed system provides the opportunity with the experiment analysis of the various deep neural network imaging models and provides proper insight for future researchers to work further in the similar systems.

#### Merits of the Proposed System

- Minimal dependency on the availability of the radiologists in hospitals (reducing the human effort).
- Usage of modern machine and deep learning techniques for better efficient and accurate diagnosis.
- Incurred cost as well as the overhead can be reduced.

### 1.3. Contributions of the Proposed Work

- This proposed work performs experimental analysis of the various image deep learning models.
- Based on the observations, we perform the comparative analysis of the model with various parameters.
- We identify the potential strengths and weaknesses of each model through the evaluation of the test samples.
- We identify and propose the suitability of these models in the corresponding areas of application based on the experimental analysis and the evaluations.

The remainder of the work is mentioned below. Section 2 describes a literature survey about existing methods used in COVID-19 diagnosis. The system architecture and its components are discussed in Section 3. Different modules of the convolution neural network and various stages of the pre-processing in detail are described in Section 4. Section 5 discusses the experimental analysis. A comparative analysis of various models through various factors is presented in Section 5. Section 6 describes the challenges, research findings. Finally, Section 6 provides the conclusion with future directions.

## 2. Materials and Methods

### Literature Survey

Zhang et al. [1] discussed the implementation of a framework which takes in the input as chest X-ray image data. This framework rendered an accuracy of 96.0% for detecting coronavirus-positive cases and an efficiency of 70.650% for coronavirus-negative cases. Wang Yunlu et al. [2] discussed a novel way for carrying out the mass screening of individuals who had unintentionally contracted coronavirus. This is used to differentiate the breathing patterns. An original, fresh, and reliable respiratory simulation (RS) model is presented in this work to bridge the space of an enormous prepared dataset and insufficient real data from current reality, which includes the characteristics of respiratory symptoms. They used bidirectional neural networks such as the gated recurrent unit (GRU) network and attentional instrument (BI at GRU) to detect six clinically important respiratory instances (Tachypnea, Eupnea, Biots, Cheyne-Stokes, Bradypnea, and Central-Apnea). With 94.5%, 94.4%, 95.1%, and 94.8%, the testing findings reveal six distinct respiratory patterns. Jiang Z Jiang et al. [3] proposed a mechanism to monitor the health of those who wear coverings by observing the respiratory system’s characteristics. This gadget combines an Android handset with FLIR (forward-looking infrared) technology and a thermal imaging camera. Pre-separating foundations and clinical objectives helps to identify those potential coronavirus infected patients in real situations. They used a combination of warm and RGB recordings from deep learning design-based cameras to conduct the wellbeing assessment in this study. They first used aspiratory information investigation strategies to identify people wearing masks; then, they used a BI at GRU work on pneumonic infection results to obtain the well-being screening result; and finally, they achieved 83.7 percent precision in identifying the respiratory medical issue of a sick patient. Imran Ali et al. [4] developed a mobile application-adaptable screening method for COVID-19 differentiation that is based on artificial intelligence (AI). In two minutes or less, the portable COVID-19 App updates the AI-based mists in the cloud that produce hack noises. A hack is typically a crucial symptom of more than 30 illnesses connected to non-COVID-19. If hacked alone is used to study COVID-19 infection, it becomes a staggeringly difficult multidisciplinary problem. A accuracy of 88.76% is achieved when morphological bearing changes are examined with differences from hack respiratory.

Brown Chloe et al. [5] suggested developing Android or iOS applications in order to gather coronavirus sound data over the publicly available sound breathing pattern of around 200 coronavirus benefits out of 7000 new clients. The authors discussed various generic ranges and three important sets of coronavirus applications with the insight of hack and breath sound. Here, the distinctions between COVID-19 and normal patients, as well as between COVID-19 -positive and non-COVID-19 asthmatic patients, are made, accomplishing 0.8 accuracy for around 200 patients. Another method which combines hack and breath rendered 82% closeness for around 30 patients. Hassan Abdelfatah et al. [6] developed a framework to assess coronavirus patients with a recurrent neural network model. This work highlighted the importance of the recurrent neural network for SSP to specifically identify the illness. LSTM was employed to evaluate the acoustic characteristics of the patient’s voice, respiration, and hack during the early screening and diagnosis of the COVID-19 infection. The accuracy of the model was poor from the evaluation of test cases on the hack and breath data of various patients. Serrurier et al. [7] used the “COUGHVID” publicly published dataset for the COVID-19 side effect hack research. More than 20,000 hacker accounts are publicly sponsored, reflecting a wide range of point orientation, age, and geographic areas. To prepare the classifier, they accumulated a succession of 121 hack noises and 94 no-hack sounds, including voice, laughter, stillness, and various foundation cries. They used self-detailed status elements (25 percent of recorded sounds had solid qualities, 25 percent had COVID-19 values, 35 percent had suggestive worth, and 15 percent had an unrevealed status). The percentage of participants who tested positive for COVID-19, those who had COVID-19 symptoms, and those who were in good health were 7.5 percent, 15.5 percent, and 77 percent, respectively. To extract tests from non-COVID-19 and COVID-19, Alsabek, M. B. et al. [8] suggested a large model that combines mel-frequency cepstral coefficients (MFCCs) and SSP (speech signal processing); it tracks down the individual link with the obtained values of the coefficients of the relationship. The results provide similarity between different respiratory sounds and coronavirus hack sounds in MFCC, despite the fact that the MFCC discourse is stronger between COVID-19 samples and samples that do not include COVID-19. Additionally, these findings are transient, and coronavirus detection allows the possibility of the removal of the voices of patients for the research. With the obtained sound samples from the Zulekha medical center in Sharjah, they were able to obtain information about COVID-19-contaminated patients. During the process of capturing the flags of discourse, the patient must be sitting with their head straight in a peaceful manner. A mobile phone is used to gather the information which contains three different accounts for the speakers. This record affects the nature of the sound. The voice information is hacked down from individual speaker.

In general, most COVID-19-infected patients have sustainability in the egregious weakness of their breathing capabilities. Mahmoud Al Ismail [9] developed a model to detect coronavirus infection through the vocal folds. The authors speculated that by looking at the progressions on the folds of the vocals, the marks of the COVID-19 infection could be discerned. The intention was to corroborate this theory and numerically illustrate the developments noticed to support the discovery of coronavirus infection over voice signal. The authors constructed vocal overlay swaying designs from recorded speech using a robust framework model for vocal crease wavering and the recently developed ADLES computation. On an experimentally chosen dataset, trial findings on COVID-19 positive and negative participants displayed distinctive examples of vocal overlap motions connected to COVID-19. For this investigation, Merlin Inc., a private company in Chile, used information collection that was clinically managed. The collection included reports of 512 individuals who had COVID-19 testing, with either optimistic or pessimistic results. This observation was performed on 10 females and 9 males. Five women and four men were tested for the coronavirus; the others were negative. The productivity of deliberate relapse on widened vowels and their mixtures is 91.20 percent. Several COVID-19 hack recording datasets have been compiled by various groups, and these datasets have been used to create AI models for COVID-19 recognition, according to Chaudhari Gunavant’s [10] research into publicly supported hack sound examples that have been gathered globally on mobile devices. These datasets also come from a variety of sources, including the extraction of information from public media interviews and clinical condition information gathering. When combined with the COVID-19 infection report, that had been applied for the AI calculation, it correctly predicts coronavirus disease ranging from 77.1% region of convergence accuracy (75.2–78.3%). Additionally, this AI calculation can add up to clinical samples from South Asia and publicly supported samples from Latin America without really planning to use the relevant cases. To identify the COVID-19 side effects, Laguarta Jord [11] built a model across the sound account hacks. It enables a solution to pre-screen the samples of the sound without incurring national expense. It successfully predicts the COVID-19 positive side effect from hack noises with 97.1% accuracy and distinguishes asymptomatic cases in light of the hack hints of 5320 chosen datasets with 100% exactness. As a result of the presence of COVID-19 side effects, such as the upper aggravation versus lower respiratory irritation plot, Quartieri Thomas [12] developed a system that comprises voice processing and demonstrating policies. It needs the intricate synchronization of neuromata by detecting the sound of the breathing systems inside of the various processes involved in respiratory systems. Pre-openness of the COVID-19 (pre-COVID-19) and post-COVID-19 are well-developed verifications that are provided by the expert evaluation with the voice gatherings of five patients. This suggested method presents a possible limit in terms of flexible and continuous evaluation to demonstrate the patient movement components, taking into account settings for preemptive counseling. Jing Han et al. [13] presented an inquiry of the coronavirus-related information by taking into account four boundaries: (i) rest quality, (ii) seriousness, (iii) uncomfortability, and (iv) uneasiness. The researchers and analysts from Cambridge University and Mellon University mailed off the “Crown voice identify App” and “Covid sounds application” used by the writers to gather data. Following information handling, these people obtained 378 complete pieces; from this basic evaluation, they selected 260 accounts for further analysis. These 256 sound samples were collected from 50 COVID-19-infected patients; for later review, the driving forces are switched over at a speed of 0.016 MHz. ComParE and eGeMAPS, two acoustic capabilities that both achieved 69% precision, were taken into consideration in this assessment. In this proposal and subsequent investigation, Kota Venkata Sai Ritwik et al. [14] looked for indications of the COVID-19 sickness. In order to extract the key points of the COVID-19 discourse from the norm, a two-class classifier was used. The small amount of YouTube video data that were collected demonstrated that an SVM classifier can achieve 88.6% exactness and 92.7% F1-score on this dataset. Further research revealed that the two classes can be distinguished from the rest by particular phone classes more effectively (stops, mid vowels, and nasals). In order to study COVID-19, Wang et al. [15] developed a non-proprietary model and comprehended sizable reference data known as COVID-19 X-rays of 13,975 patients. This approach not only promised more notable experiences of COVID-19 fundamental elements, but also distinguished significant information from the examined images. Their analysis produced an accuracy of 93.3% for the COVID-19 dataset. X-beam and CT filter image datasets were subjected to Maghdid’s method [16] thorough convolutional neural network (CNN) application and the use of AlexNet. They identified 94% accuracy (particularly 88%) for CNN and 98% accuracy (explicitness 96%) for AlexNet. CNN-based techniques were applied by Apostolopoulos and Mpesiana to a small dataset of clinical images. A total of 1427 X-beam photos, including 224 COVID-19 images that were confirmed, were included in their dataset. They each had the highest levels of explicitness (96.46%), awareness (98.66%), and precision (96.78%). In their COVIDX-Net proposal, Hemdan, Shouman, and Karar [17] analyzed 50 chest X-beam images with 25 COVID-19 cases using seven unique profound CNN models. They obtained a 90% accuracy and 91% F-score from their calculation. Using X-beam and CT filter images, Islam M. et al. [18] conducted a number of tasks using deep learning approaches to distinguish COVID-19 patients. Using sophisticated learning algorithms, their calculation contained contaminated districts and determined the starts to finishes for COVID-19. Additionally, they used inception residual recurrent CNN with transfer learning and obtained 98.78% precision in CT pictures and 84.67% testing exactness from X-beam images. In a similar work, [19], the authors combined image processing and ML over the X-ray and CT-image datasets, and the accuracy range was from 89 to 99%. The electronic medical records (EMR) of a patient are confidential data which cannot be accessed, and machine learning algorithms require data for training their model. The federated machine learning model renders an effective solution while dealing with data privacy. Abdul Salam et al. [20] discussed the efficiency of federated learning by analyzing chest X-ray images from COVID patients in Keras and TensorFlow models. COVID-19 has affected us severely, and one of the protective ways is to wear a mask in public. Mohamed Loey et al. [21] discussed a hybrid model which combines deep and machine learning techniques for face mask detection. The feature extraction is performed using resNet, and the classification process is performed by implementing decision trees, support vector machine (SVM), and the ensemble algorithm. Different datasets were used for the training and testing of the model, and the authors concluded that the SVM classifier achieved the maximum accuracy. In a similar research, Rehman A. et al. [22] and Sweta Bhattacharya et al. [23] reviewed the possibilities and challenges of combining DL and ML techniques for COVID-19 detection. Kwekha-Rashid et al. [24] studied the implications of applying various ML applications over the COVID-19 dataset. Upon extensive survey, the authors concluded that supervised learning produced better accuracy than the unsupervised learning algorithms.

## 3. System Architecture

**Figure 1:**
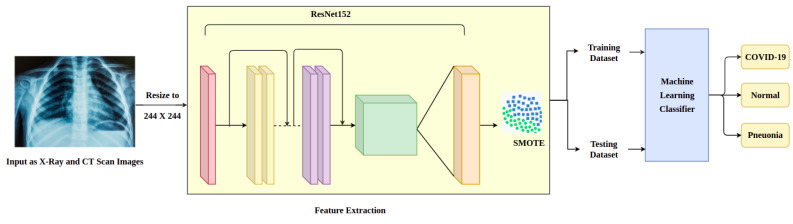
Architecture of the proposed system with a sample scenario of Resnet model.

The above architecture shown in Figure 1 describes the learning process of the deep neural network with the input, hidden layer and the output layer. The output layer is connected to the SMOTE (synthetic minority oversampling technique). This technique allows the increase in the number of instances in a dataset in a balanced way. This is a statistical modeling technique in Python. After the training, the architecture is ready to analyze the chest X-ray images and classifies them into three major classes, such as the COVID-19-infected, pneumonia-infected and normal images. The chest X-ray images contain more features; these features train the system using the deep learning neural networks. The various models used in the work are Inception V3, VGG16, Xception, ResNet, CNN and ResNet with VGG. Based on the correctness of the classification of the image in three major categories, including COVID-19, non-COVID-19 and pneumonia, we determine the accuracy of these deep learning models. Based on the training efficiency, the accuracy and loss values of the above-mentioned models can be evaluated as normal or the fluctuating values. The above architecture provides one such test case with the ResNet model.

### 3.1. Module Description

#### 3.1.1. Data Pre-Processing and Augmentation

Each image must be pre-processed according to the deep brain network that is being used. There were two major advancements: resizing and standardizing. As suggested by their described architecture, different neural networks require images of differing sizes. ResNet18, DenseNet121, and MobileNetV2 demand images to be 224 × 224 pixels in size, but InceptionV3 and Exception require images to be 229 × 222 pixels in size. The individual models standardize each of the images as well. A sufficient preparation of a neural net necessitates a large amount of data. Increased access to information addresses this problem by making the best use of the current data. It helps the model avoid over-fitting the current dataset by allowing it to grow in size (usage of SMOTE).

#### 3.1.2. Convolution Neural Networks

In 1989, CNN was first used for transcribed postal division acknowledgment. This network follows the feed-forward mechanism. The primary advantage of CNN compared with the predecessors is the capability of detecting significant pieces with little to no human intervention. On the information image, a series of convolution and pooling activities are performed, followed by a single or more fully related layers. The resultant layer is dependent on the tasks that are being completed. The result layer for multi-class characterization is a softmax layer. The most significant challenge with more depth CNNs is vanishing slopes, which may be overcome by leveraging the lingering networks described in the next section.

#### 3.1.3. Transfer Learning

A model built for a single task is used as the starting point for the learning process and then applied with transfer learning for multi diversity. As a result, rather than going through the protracted process of preparation with arbitrarily introduced loads, pre-prepared models are used as the starting point for a few clear tasks in transfer learning. As a result, it aids in the preservation of important assets required to build neural network models to address these issues. To improve the understanding of exchange learning, researchers employed space, task, and minimal probability to suggest a framework. Preparing a deep learning-based model for clinical finding-related concerns is computationally expensive due to a lack of a suitable dataset, and the results achieved are also insufficient. In this work, pre-constructed deep learning models were used, which were recently prepared on the ImageNet dataset. This plethora of pre-made models was also calibrated for pneumonia order [25].

#### 3.1.4. Training Images Using an Algorithm

The fundamental advantage of CNN in comparison with its predecessors is that it is capable of identifying crucial highlights with little or no human intervention. ConvNets are more spectacular than AI computations, and they are also more computationally efficient. In light of their identified properties, these mathematical traits are subsequently grouped into mathematical clusters. These exhibitions are then put in various hubs around the organization and subjected to various levels of focus depending upon the information provided. The CNN models are used for the topographical characterization in a variety of organizations that require information to be organized quickly and securely. It almost acts as a conduit, removing residue and separating the image parts. The image data are subjected to a series of convolution and pooling operations, which are followed by single or several totally related layers. The resultant layer is dependent on the actions being carried out. A random seed is fixed during the preparatory interaction to make the results repeatable and compelling. For the COVID-19 disease location, the feature order is used. The harmful materials are removed from the human body and replaced with sound lung images. When the lungs are healthy and there is no order, the results are solid, but when there is a disease that produces dark spots on a dim scale, it reveals the type of infection and its characteristics. A couple of clusters with mathematical modeling come together to form a group. Depending on the dataset provided, it is either a sound or an infected lung if the mathematical clusters match. Grouping is a simple but crucial method that produces a valid result and is used to implant infected sites.

## 4. Results

There are various deep image neural networks that are compared and analyzed with the test chest X-ray images against the trained neural network models. The different models under study are discussed below.

### 4.1. Resnet with VGG

This section explains VGG with Resnet. The current approach correctly identified 52.56% of normal cases and 94.02% of infected cases. However, the proposed model identified normal cases 69.66% of the time and infected cases 98.29% of the time. For the normal cases, this algorithm performs 17.10% better and for the infected cases, 4.27% better.

**Figure 2:**
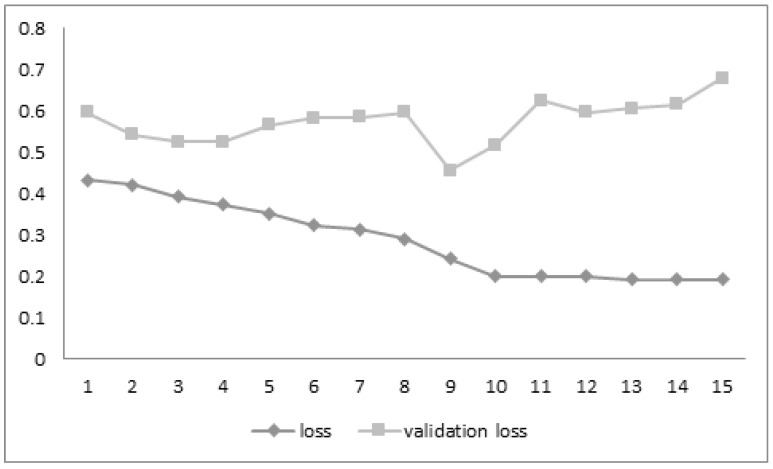
Graph comparisons for the loss vs. validation loss parameters.

**Figure 3:**
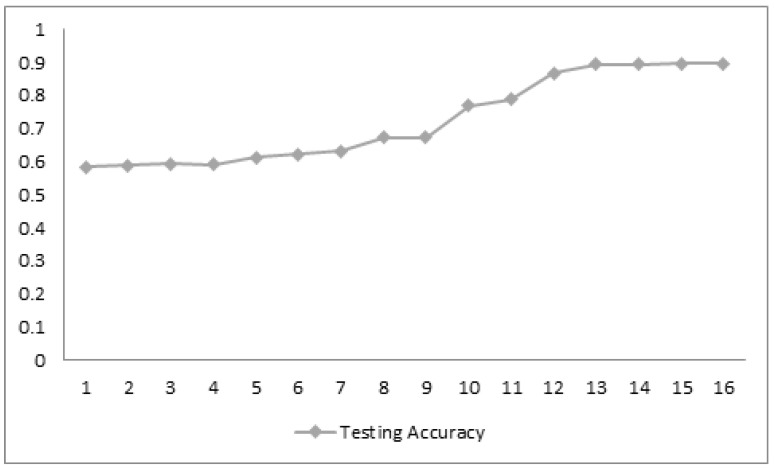
Evaluation of the testing accuracy.

**Figure 4:**
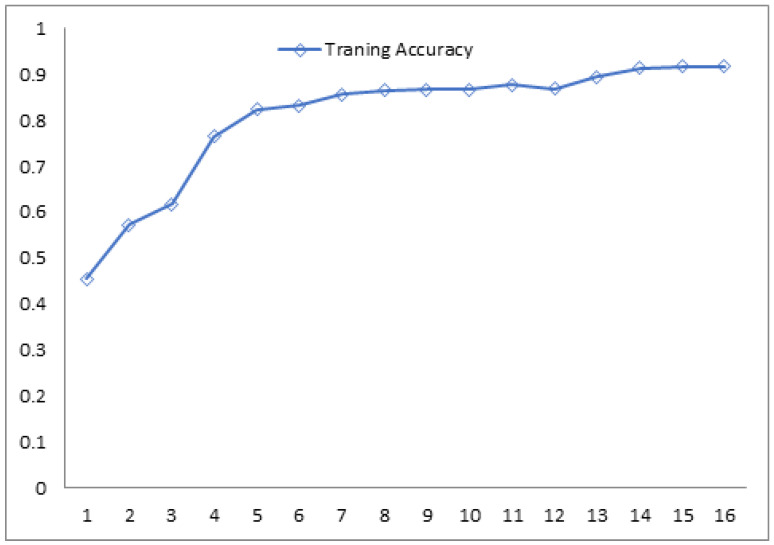
Evaluation of the training accuracy.

The loss function gradually decreases with the increase in the training epochs, but the validation loss is variable and unstable through the sample analysis process. The efficiency of the training and testing algorithms increase gradually, as there is an increase in the epochs. This shows the possible alternatives for the measurement of COVID-19-affected images and non-COVID-19 images during the preliminary analysis of the dataset. Figure 2 and Figure 3 show the gradual increase in the training and testing efficiency, as the number of epochs for the training sample increases the accuracy function, which is in general measured between a value varies from 0 to 1, as the training and testing epoch sample reaches around 1–15. The value of both training and testing accuracy almost becomes closer to 1. Thus, these factors indicate that the pre-processing is successful and also the training efficiency and the testing efficiency of the proposed system are improved through pre-processing and sample analysis of the dataset. The loss function also diminishes, but the validation loss is non-uniform, even if there is an increase in the training accuracy, shown in Figure 4, and the testing accuracy.

This could be a challenge during the model evaluation and model analysis process and can affect the accuracy and F1-score under measurement. In the next step, we develop the models through definition, initialization of attributes and evaluation. In the next section of the paper, we perform the comparison analysis of the models with relevant attributes required for the comparison with final prediction of the suitability of the same. The results obtained for each epoch is tabulated with relevant parameters in Table 1.

### 4.2. Convolutional Neural Networks

The dataset is validated into three classes, which are normal, pneumonia and COVID-19. These classes were trained with 175 samples per epoch, and 10 such epochs were analyzed for the performance of various factors of measurement, which are tabulated in Table 2. Although the training accuracy dropped a bit toward the end, the validation accuracy remains constant. The loss function is reduced during the training process, but the validation loss was variable throughout the training process. The other techniques for training image datasets must be evaluated before making a conclusive remark on the final prediction of the suitable training and modeling algorithm. The convolutional neural network is the fundamental solution for the COVID-19 X-ray analysis. The other techniques and their implementations will be discussed in the successive sections.

**Figure 5:**
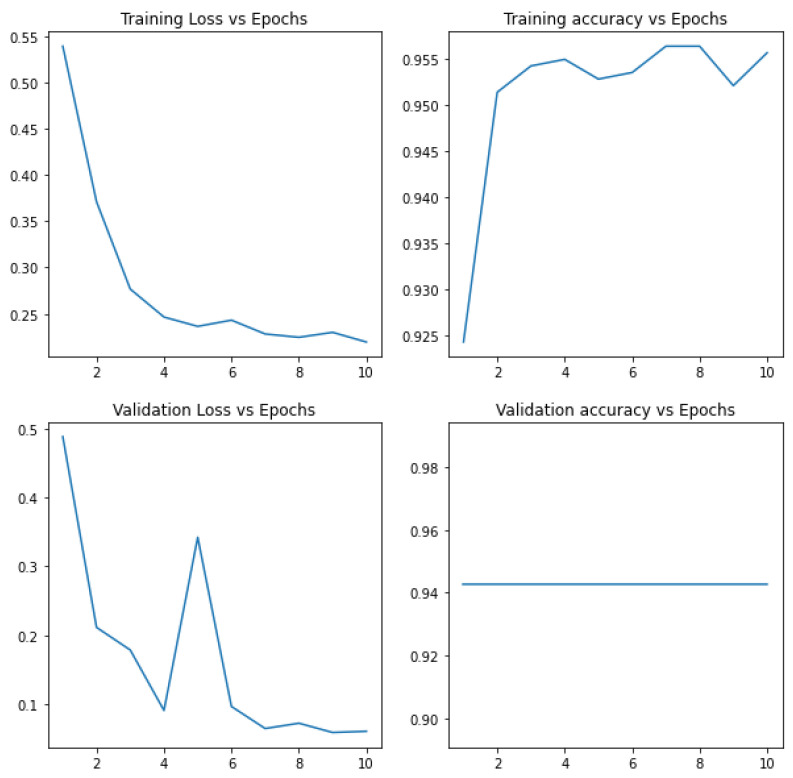
Evaluation of the training and testing accuracy for CNN.

Figure 5 shows the response of the CNN model for training, and the loss is a gradient function here, which is unpredictable. This makes it unsuitable for scientific and clinical research applications. Figure 5 shows the analysis for validation loss and validation accuracy. The validation accuracy is constant, and the validation loss is variable, making this model unsuitable for critical applications.

### 4.3. Xception with Transfer Learning

Xception with transfer learning uses 30 epochs, and each epoch processes 75 samples each; the final tabulation is listed below in Table 3. The accuracy achieved at the end of the 30th epoch is 97.16%. This model provides better accuracy compared with the CNN, and it produces the result with fewer iterations. This is due to the transfer learning of Xception learning, which provides enhanced accuracy with fewer iterations. The behavior of the model is much better compared to CNN since the loss function decreases gradually as the training increases. The results are tabulated, and the figures are plotted below for the accuracy and loss function.

**Figure 6:**
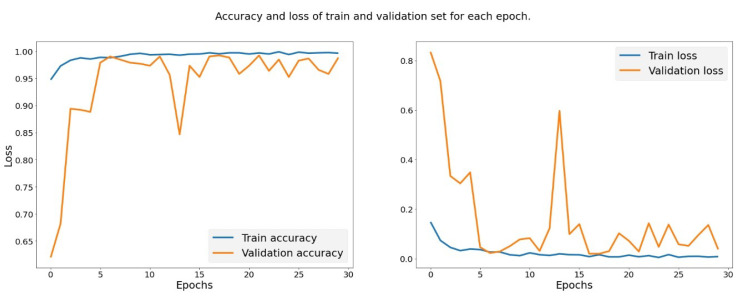
Evaluation of the training accuracy for Xception model.

It is evident that both the train loss and the validation loss decrease as the number of epochs increases, and training and the validation accuracy increase when the number of epochs increases. Table 3 shows the result analysis of the Xception algorithm with the transfer learning with various factors of measurement. The results in Figure 6 show that there is a gradual increase in training and testing accuracy and a systematic decrease in training and the validation loss. This predictable and trusted nature of the result makes the Xception suitable for the imagery application with the minimum trusted performance, and the behavior of the model is guaranteed to deliver the desired performance without much deviation.

### 4.4. VGG16 Model

Table 4 presents the result analysis of the VGG16 deep image learning model. The VGG16 algorithm provides 200 epochs for the training of the model. The result analysis is performed for the learning accuracy, loss, validation loss and F1-score. The value of accuracy is measured as 93%, and the F1-score is measured as 0.93.

**Figure 7:**
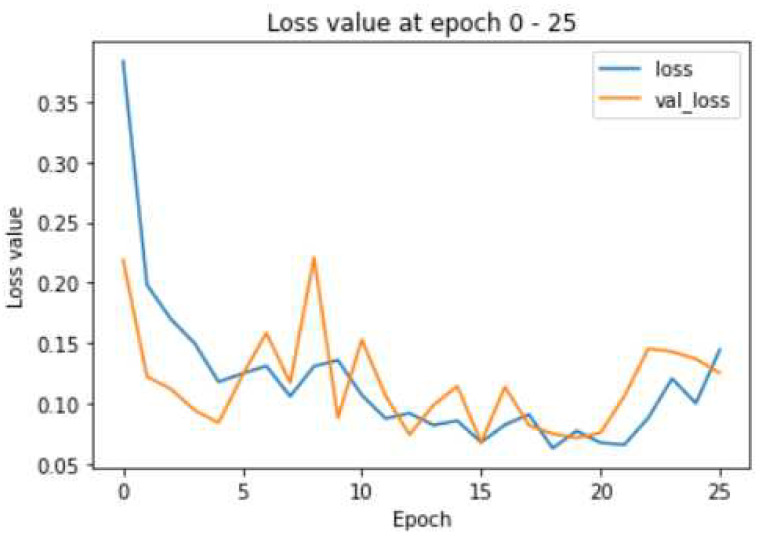

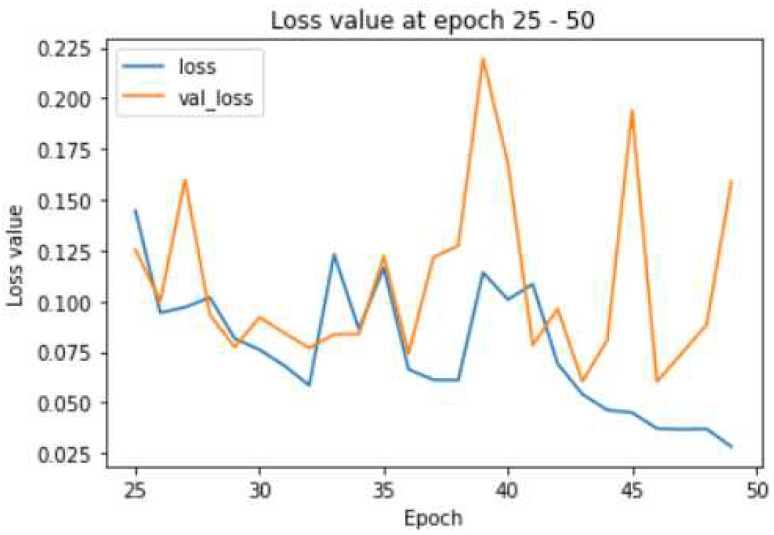
Evaluation of the loss and validation loss in VGG16.

**Figure 8:**
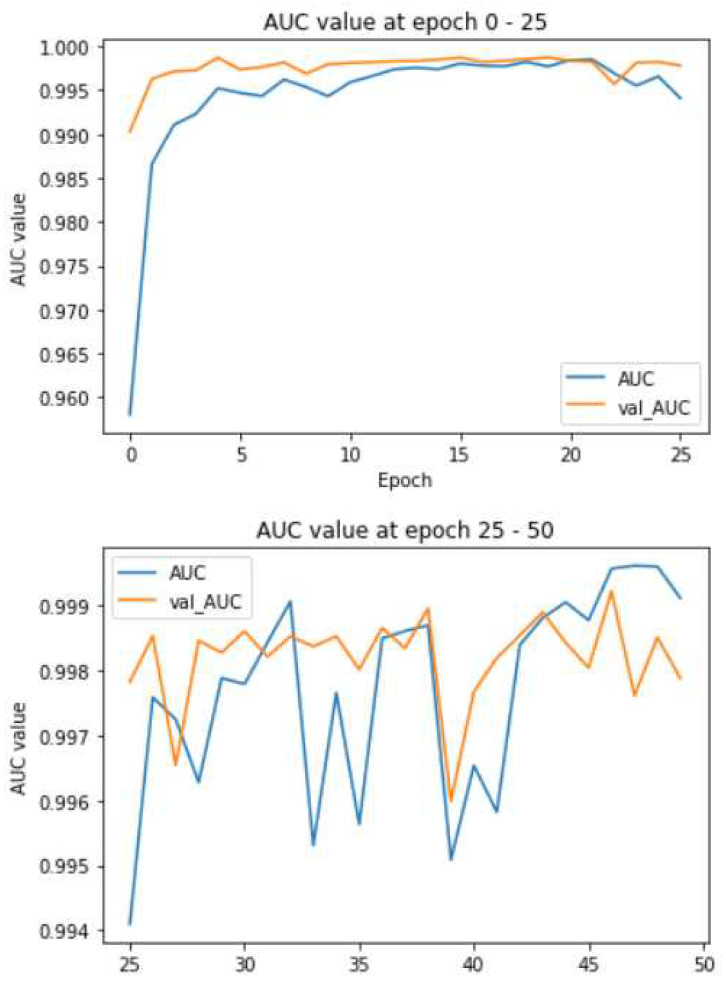
Evaluation of the training and testing accuracy in VGG16.

The accuracy plot increases gradually, but the loss function takes a spike at intervals during the training process. Figure 7 shows that the loss function of the VGG16 algorithm. This model is not suitable for the research or any other critical applications since the validation loss shows a major spike in the middle. However, the generic loss uniformly decreases with the increase in the training samples. Both the training and validation accuracy increases with the increase in the training process, which is also evident from the Figure 8. Thus, this model is suitable for the experimental research but not for the scientific critical research and development.

### 4.5. ResNet

Table 5 shows the result analysis of the ResNet model with 50 epochs. This uses 34 sub-samples for the analysis of the training efficiency and the loss function for training and validation. The accuracy is measured to be 82% with an F1-score of 0.82, which is lesser when compared with all other types of image classification techniques. The validation accuracy increases constantly, and the validation loss is variable here. Training loss is negligible.

### 4.6. Inception V3

Table 6 shows the result analysis for the Inception V3 algorithm with 15 epochs and 148 samples per epoch. This model performed with 15 epochs with 148 samples processed at each epoch. This model provide the highest amount classification accuracy at around 0.9688, with an F1-score of 0.9688.

This model may be preferred for research labs, engineering projects, etc., since the accuracy is greater, and it can perform the classification with the desired accuracy as per the need of the target application. For visualization of the classification process, we may prefer ResNet with VGG or Xception models.

The detailed descriptions about the various models used in deep image neural networks are discussed below in Table 7. The models of the convolutional neural network for deep image neural modeling are termed into a broad category called Imagenet, which was earlier developed in 2012. Earlier Alexnet was developed in 2012, VGG and Inception were developed in 2014, and Resnet was developed in 2015. Resnet has only a 3.6% error rate, which is less than all the models. The models of Imagenet are trained with 1.2 million images with 50,000 images for validation and 100,000 images for testing. The goal of image net is to define 1000 image object features for an image that is used in day-to-day life. The main hyperparameters for tuning these models are F1-score, training and validation accuracy, and training and validation losses, which are measured and tabulated above. The comparative analysis of all the features is discussed in the next chapter.

## 5. Discussion

### 5.1. Comparative Analysis of the Various Deep Neural Network Models

Various algorithms, such as Inception V3, Xception, VGG16, CNN, ResNet and ResNet with VGG, are tested for the comparative analysis. The results are obtained for four major parameters such as precision, recall, F1-score and support. The comparisons are plotted for the above-mentioned algorithms in a graph, taking these parametric values into the Y-axis with the corresponding comparison factors in the X-axis. In the above-stated algorithms, ResNet with VGG provides the highest accuracy with the fewest epochs (15 epochs). This method provides the best of all accuracy and the F1-score as 0.9827 for the infected cases. However, for normal cases, it offers an accuracy around 0.6966. However, the overall accuracy drops because of the loss of accuracy in the detection of the X-ray of the normal cases. Considering the second algorithm CNN, the overall test samples were run and provided an accuracy of around 0.9586. However, here, the loss function is very minimal compared to the other methods, such as 0.1102. This method is preferred in cases that we need decent accuracy with minimal loss. The next experiment analysis was performed for the Xception model, which provided the accuracy levels of 0.92 and F1 score around 0.94 with minimum training loss. The next model applied for analysis was VGG16. This model ran around 200 epochs of 34 samples each and provided an accuracy of around 0.93 with an F1-score of 0.93. This method also reduced the training and the validation loss, as the number of samples is sufficient to estimate the results in 200 epochs. Even though the iterations for this model are time consuming, it provided a linear reduction in the loss function as the training of the samples increased. The next method applied here is the ResNet, which offered non-linear behavior of the loss function. However, the accuracy did not become reduced, and it was poised at 0.83 during the end of the evaluation of the test samples. Compared with the other models, Resnet offered the worst performance in the above-said environment. Finally, Inception V3 provided the best overall accuracy through the sample analysis of 15 epochs with 148 samples. This model provided 96.88 and with an F1-score of around 96.88. While other models may be preferred for the imagery, Inception V3 may be preferred for model evaluation, analysis, research companies, etc. As an outset, resNet with VGG is the best companion for the detection of the COVID-19 virus. In the case that the training samples do not get updated, Xception is the most suitable for the processing technique for the image dataset. As per the accuracy, Inception V3 is preferred, and as per the imagery, resNet with VGG and Xception are mostly preferred. The comparison analysis is performed for the algorithms; the results are listed below in the comparison chart which brings out a clear idea about the model accuracy, loss function and F1-score, based on which we hand-pick one algorithm based on the requirement of the end user.

**Figure 9:**
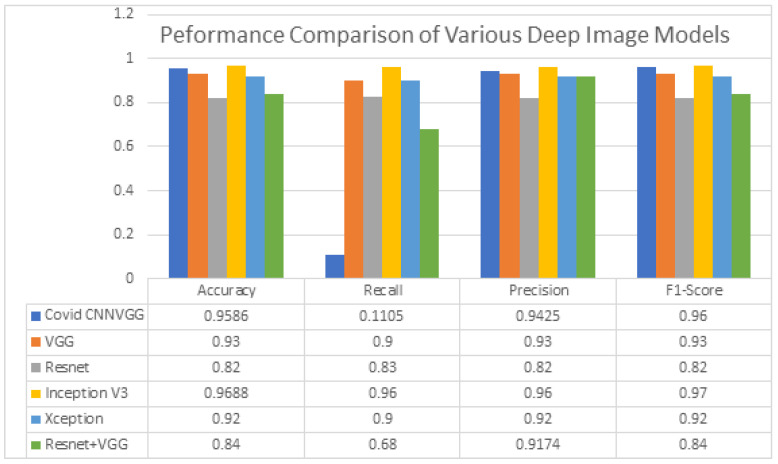
Comparative analysis of all the models of deep neural networks used for COVID-19 X-ray imaging.

Figure 9 shows the performance comparison of the COVID-19 detection deep learning models. This comparison model takes four parameters to compare the efficiency of each deep learning model based on the values recorded under each parameter. With respect to the metric accuracy, Inception V3 outperforms all models, with an F1-score of 0.97 and accuracy of 0.9688. So, this model is critical for scientific research and experimental implementation purposes. CNN comes next with 0.9586, and the rest follow. With respect to the metric precision, again, the Inception V3 and CNN take a leading step that confirms the possibility of the scientific and research application of these algorithms. The recall factor is better in Xception, and it is hence preferred for the imaging applications. The F1-score is best in both CNN and Inception V3. Looking at various categories, we observe that CNN and Inception V3 may be the most preferable for the scientific research and implementation and for the deep neural networks for the creation of deep learning models for the COVID-19 detection scheme through X-ray imaging. The best of all is Inception V3. Authors should discuss the results and how they can be interpreted from the perspective of previous studies and of the working hypotheses. The findings and their implications should be discussed in the broadest context possible. Future research directions may also be highlighted.

### 5.2. Challenges of the Existing Systems

The following challenges arise during the evolution of the COVID-19 X-ray imaging systems.

#### 5.2.1. Variation in Mutation of COVID-19 Infection

The COVID-19 virus mutation is the biggest threat to the medical world since it takes different mutations across different places of the world. Some variations are epidemic, and some variations are pandemic. The pandemic variations cause severe damage to the human community. In 2022, we experienced a range, from the BA-1 to BA-4 types. Fortunately, these types are not very life threatening. When the mutation happens, the virus changes its DNA and protein structure and changes into an entirely different genetic formation. This is the biggest challenge for scientists, doctors and health specialists. This would also be a biggest challenge to the medical imaging research since the virus changes its dimensions very frequently.

#### 5.2.2. Regions of Impact of COVID-19 Virus in the Human Body

The previous delta variants of the coronavirus were affecting the lower part of the chest, causing severe congestion, damage of the lungs and pneumonia fever by compromising complete lung function, causing mortalities around the world. However, the later minor variants of the BA type viruses did not create any serious impact on the lower part of the lungs, since they attacked only the upper part of the lungs, without much causalities or compromise to the lung function. However, as the imaging perspective, the density of the infection is almost absent in the lower lung, which creates serious changes in the X-ray patterns and further analysis in the image acquisition systems. This would increase the demand for further training of the model with variations in the infection patterns, thus making the re-training of the deep learning models by increasing the complexity.

#### 5.2.3. Training Efficiency

Since the mutation of virus and the variation of its impact on human body, the variations required for the imagery analysis become an inevitable factor. The exhaustive re-training is required in existing deep image neural networking models. This increases the time complexity. Some models do not guarantee optimal efficiency and accuracy during the training process. They often exhibit inconsistent behavior during the measurement of validation loss and training accuracy (CNN). Thus, increasing the training samples or re-training of these systems would pose a great challenge to the existing models which are used right now. So, new weight distribution schemes may be required when the mutation of the virus acquires a major change in the structure.

#### 5.2.4. Hardware and Software Requirements

The evolution of the virus demands updates in medication, imaging, and the training of models. It also requires updates of the existing hardware and software to understand and support the variation of the mutation by increasing the processing capacity of the GPU, TUP processors, storage support and processing support of the software with proper updating and maintenance. The performance of the models is also based on the availability of the processing memory and the capacity of the processors. The vendors of these hardware and software should identify contemporary design strategies and techniques that provide the utmost support for the smooth processing of these models when the re-training takes place. Thus, the change in the hardware and software requirements is inevitable for the change in the virus mutations.The below listed elements are the research findings acquired through this experimental analysis and comparisons performed on the previous chapters of this proposed work.

### 5.3. Research Findings

- The resNet+ VGG and VGG show the sudden variations in the validation loss and hence make it unsuitable for research and critical application related to medical imaging. In general, fluctuations in the dataset or improper design of the neural architecture are the major reasons for the variation in loss and training efficiency. This may be also due to the overfitting of the algorithm and because of the increase in the number of layers compared to the original layers required. When we analyze and observe the above-mentioned factors, we can improve the training efficiency and reduce loss.
- CNN also is not very preferable for critical applications since both the training accuracy and validation loss are unpredictable. The training accuracy may be affected due to the above-said reasons, and also due to the improper assignment of the learning rate. The inadequate learning may develop inconsistencies in the training model, which in turn affect the training and testing of the models by reducing the training and testing accuracy to the significant levels.
- ResNet is not so preferable because of the low accuracy and the F1-scores. A higher level of accuracy is required to predict image data, and hence, the size and the number of features relevant to an image data are very high. The dimensionality variations and up- or down-scaling may be required for these images when we perform training or sample analysis of the dataset. When we change such factors, the required classification accuracy for the algorithm is required to be very high. Thus, we define that the ResNet may not be preferable for imagery applications. This can be improved by re-training the model to the next level.
- Inception V3 is preferable for scientific research and critical applications, since the accuracy and the F1-scores are high. Since the values obtained are accurate, this algorithm ensures the proper application of the dataset in the imagery applications related to medical imaging. For clinical and medical research, if the application needs more criticality, then using Inception V3 is the best solution.
- Xception is preferred for the imagery applications because of the consistent performance of the accuracy and the loss functions. The image dataset is huge and continuous. The training model requires more image vectors for training. These vectors and features need to train the model for a relatively longer duration of time. For any system that uses the medical data for training purposes, the response of the system is required to be uniform, stable and predictable. Xception is one such algorithm that has a higher stability and uniformly increasing function of accuracy as well as a decreasing function of loss.

## 6. Conclusions

Because of the COVID-19 pandemic, profound learning plays a significant impact, developing the possibility for accurate judgment and response to the outbreak. The work observed the scientific and predictive potential of profound observations on lung radio-graphs with the presentation of a picture classification technique based on the COVID-19-Net to classify chest MRI images. The approach focuses on exchange learning, model combining, and identifying three types of chest MRI images: ordinary, coronavirus, and viral pneumonia. The selection of models ResNet and ResNet with VGG have a significant effect on the combination of precision and loss functions, gradually increasing their proportion of neural weight during the preparation cycle. With Inception V3, the model predicted COVID-19 conditions with 96% for the MRI images acquired of the chest. It serves as a resource for research critical applications health institutions, medical offices, government institutions, and, shockingly, the global conclusion of the COVID-19 pestilence condition. Every day, the coronavirus pandemic is becoming more complicated, with a rapid increase in the number of COVID-19 cases. Hence, fast mass COVID-19 testing may be necessary. This proposed work provides various measures and provides numerous options for using CNN models to classify COVID-19-affected patients based on the scan of chest imagery using MRI. Furthermore, we assume that in the available versions, the Xception net provides the best-of-all display, and is the most suitable for the purpose. We successfully organized coronavirus sweeps, demonstrating the potential for using such technologies to automate the concluding of tasks in the near future. The factor may be evaluated by comparing it to fresh information that has recently become available. Later on, we may use the massive dataset of the MRI of the chest for the validation of our suggested model. It is also recommended that any effective use case of this proposed work could be discussed with clinical professionals.

## Figures and Tables

**Table 1:** Parameters of the Resnet with VGG experimental analysis.

Loss	Validation Loss	Training Accuracy	Testing Accuracy
0.432	0.59632	0.5843	0.454724
0.42123	0.54212	0.58772	0.5723
0.39224	0.52479	0.59431	0.617332
0.37233	0.52556	0.59213	0.7643
0.352322	0.565712	0.61212	0.82433
0.32241	0.583232	0.6221	0.832435
0.312013	0.583632	0.63211	0.85674
0.29012	0.59632	0.67321	0.86543
0.241009	0.45632	0.6731	0.8667
0.1997	0.51632	0.7683212	0.8665
0.1992	0.62532	0.788276	0.87665
0.198892	0.59632	0.86843	0.86789
0.19222	0.60541	0.893271	0.89445
0.19123	0.615931	0.8932	0.9133
0.191232	0.679632	0.8974	0.91776

**Table 2:** Result Analysis of CNN.

Parameter	Values
Accuracy	0.9586
loss	0.1105
Validation loss	0.2133
Validation accuracy	0.9425

**Table 3:** Result analysis of the Xcpetion model.

	Precision	Re-Call	F1-Score	Support
Normal	0	0.8	0.88	87
Pneumonia	1	0.99	0.94	101
Accuracy			0.92	188
ROC-AUC Score	0.895299145			
F1 Score	0.937880633			

**Table 4:** Result analysis of the VGG16 model.

	Precision	Re-Call	F1-Score	Support
Normal	0	0.80	0.88	87
Pneumonia	1	0.99	0.94	101
Accuracy			0.93	188
F1 Score	0.93			

**Table 5:** Result analysis of the ResNet model.

	Precision	Re-Call	F1-Score	Support
Normal	0	0.91	0.82	87
Pneumonia	1	0.74	0.82	101
Accuracy			0.82	188
F1 Score	0.82			

**Table 6:** Result analysis of the Inception V3 model.

	Precision	Re-Call	F1-Score	Support
Normal	0.92	0.99	0.96	87
Pneumonia	0.99	0.93	0.96	101
Accuracy			0.96	188
F1-Score	0.9688			

**Table 7:** Various deep learning models used with required details.

S.NO	Model Name	Description	Neural Structure	Demerits and Merits	Contributors
1	Inception V3	Multi-level feature extraction mode	It performs neural convolutions of 1 × 1, 3 × 3 and 5 × 5 before the weights are transferred to the next layer	Weights of the Inception is smaller than VGG 16 and resent	Szegezy et al.
2	Xception	Replacement of standard Inception model with depth wise separable convolutions	Same as Inception with depth-wise separable convolutions	Smallest serialization of weight	Francois Collet
3	VGG	Simple in nature with 4096, where the reduction is done by max-polling.	3 × 3 convolution layers with with softmax classification at the output	Training the model is very complex. Pre-training is required for large models. Network architectural weights are quite large	Simonyaan and Zisserman
4	Resnet	Accuracy is improved by mapping of the identity. The versions of this network are identified by weighted layers	Extremely deep neural networks trained with SGD	The model is deeper than VGG 16 but the size is very small because it takes the average of polling rather than fully connected layers	Kaimeng He

## Data Availability

Training data was collected from https://www.kaggle.com/datasets/praveengovi/coronahack-chest-xraydataset.
